# High levels of psychopathic traits alters moral choice but not moral judgment

**DOI:** 10.3389/fnhum.2013.00229

**Published:** 2013-06-04

**Authors:** Sébastien Tassy, Christine Deruelle, Julien Mancini, Samuel Leistedt, Bruno Wicker

**Affiliations:** ^1^Institut de Neurosciences de la Timone, CNRS UMR 7289 and Aix-Marseille UniversitéMarseille, France; ^2^Department of Psychiatry, Assistance Publique - Hôpitaux de Marseille, Sainte Marguerite University HospitalMarseille, France; ^3^School of Medicine, Aix Marseille Université, UMR912 SESSTIM (Economics and Social Health and Medical Information Processing)Marseille, France; ^4^Public Health and Medical Information Department (SSPIM), Assistance Publique - Hôpitaux de Marseille, La Timone University HospitalMarseille, France; ^5^Forensic Psychiatry Unit, Department of Psychiatry, Erasme Academic Hospital, Université Libre de BruxellesBruxelles, Belgium; ^6^Medical Anthropology, SPF JusticeBrussels, Belgium

**Keywords:** moral, psychopathy, decision making, judgment, choice, emotion

## Abstract

Psychopathy is a personality disorder frequently associated with immoral behaviors. Previous behavioral studies on the influence of psychopathy on moral decision have yielded contradictory results, possibly because they focused either on judgment (abstract evaluation) or on choice of hypothetical action, two processes that may rely on different mechanisms. In this study, we explored the influence of the level of psychopathic traits on judgment and choice of hypothetical action during moral dilemma evaluation. A population of 102 students completed a questionnaire with ten moral dilemmas and nine non-moral dilemmas. The task included questions targeting both judgment (“Is it acceptable to … in order to …?”) and choice of hypothetical action (“Would you … in order to …?”). The level of psychopathic traits of each participant was evaluated with the Levenson Self-Report Psychopathy (LSRP) scale. Logistic regression fitted with the generalized estimating equations method analyses were conducted using responses to the judgment and choice tasks as the dependent variables and psychopathy scores as predictor. Results show that a high level of psychopathic traits, and more specifically those related to affective deficit, predicted a greater proportion of utilitarian responses for the choice but not for the judgment question. There was no first-order interaction between the level of psychopathic traits and other potential predictors. The relation between a high level of psychopathic traits and increased utilitarianism in choice of action but not in moral judgment may explain the contradictory results of previous studies where these two processes were not contrasted. It also gives further support to the hypothesis that choice of action endorsement and abstract judgment during moral dilemma evaluation are partially distinct neural and psychological processes. We propose that this distinction should be better taken into account in the evaluation of psychopathic behaviors.

## Introduction

Psychopathy is a personality disorder characterized by emotional dysfunction, callousness, manipulativeness, reduced guilt, remorse and empathy, egocentricity, and antisocial behavior including impulsivity and poor behavioral control. Moreover, psychopaths frequently engage in morally inappropriate behavior, including taking advantage of others, lying, cheating, and abandoning relationships (Cleckley, 1941; Hare, 1999). Although psychopathy increases the probability of immoral behavior, experimental studies exploring its influence on decision making during moral dilemma evaluation have yielded contradictory results. Some studies report that psychopathy does not influence the decision (Blair, 1995; Glenn et al., 2009; Tassy et al., 2009; Cima et al., 2010) while others report that it is associated with higher probability of responses that favor the sacrifice of one individual for the greater welfare of many (i.e., utilitarian bias) (Glenn et al., 2010; Bartels and Pizarro, 2011; Koenigs et al., 2012). This latter result is consistent with the idea suggesting that emotion is a key element leading to non-utilitarian moral judgments and hence that individuals with low emotional responsiveness, such as those high in psychopathy, are expected to make more utilitarian judgment (Eslinger and Damasio, 1985; Greene et al., 2004; Koenigs et al., 2007). It is also consistent with the proposal that psychopathy tends to reduce the empathy for the victim, leading to greater concern for the mathematically rational ends than the emotionally aversive dimension (Greene et al., 2004, 2008; Crockett et al., 2010). Based on clinical experience, several authors have also reported that psychopaths are individuals with normal—or even higher—intelligence and a normal ability to judge, but whose actual behaviors remain particularly immoral (Cleckley, 1941; Hare, 1999; Glenn et al., 2010). This discrepancy between the intact ability to judge and an altered behavior suggests that these processes are at least partially independent, as proposed in the case of patients with ventromedial prefrontal brain lesions who exhibit inappropriate social behaviors but a preserved judgment ability (Eslinger and Damasio, 1985). Somewhat counter-intuitively, moral choice of action as reflected in actual behavior could thus be independent of moral judgment. Recent behavioral studies support such discrepancy by reporting experimental evidence for a divergence between judgment and choice of action during moral evaluation (Kurzban et al., 2012; Tassy et al., 2013). Moreover, moral choice of action and moral judgment could rely on partially distinct neural processes. A support to this hypothesis comes from results of a recent study showing that neural disruption before moral dilemma evaluation alters the judgment (objective evaluation) without modifying the subsequent choice of action (Tassy et al., 2012).

Psychopathy has traditionally been conceptualized in forensic samples. It describes a subset of individuals with Antisocial Personality Disorder who exhibit distinct personality features. However, recent taxometric studies suggest that psychopathy is a dimensional construct rather than a qualitatively distinct category of behavior and should be considered as an extreme variant of normal personality (Levenson et al., 1995; Hare and Neumann, 2005; Walton et al., 2008). The level of psychopathic traits would thus exist on a continuum in the general population and individual differences can be reliably assessed via self-report measures (Lilienfeld and Andrews, 1996; Edens et al., 2006). In the present study we seek to determine if a high level of psychopathic traits, in particular those related to primary psychopathy characterized by affective deficits (Karpman, 1946) and ventromedial prefrontal cortex (VMPFc) hypoactivation (Lotze et al., 2007), may be associated to an utilitarian preference in the specific case of moral *choice* during moral dilemma evaluation.

## Materials and methods

### Psychopathy scale

The Levenson Self-Report Psychopathy (LSRP) scale is the only global measure of psychopathic traits in the general population validated in French (Levenson et al., 1995; Chabrol and Leichsenring, 2006; Campbell et al., 2009). It consists of self-administered questionnaires with 26 items in a 1–4 Likert-type agree/disagree rating scale. This scale is further subdivided into two subscales.

The LSRP1 is a subset of 16 items from the complete questionnaire constructed to determine the degree to which participants report interpersonal-affective characteristics that are associated with factor I of the Psychopathy Checklist-Revised (PCL-R) and that are the hallmark of primary Psychopathy based on Cleckley's and Hare's conceptualizations of the disorder (1, 2). The 10 remaining items compose the LSRP2, which measures the traits related to the social deviance associated with the factor II of the Hare Psychopathy Checklist.

### Dilemmas

We presented 10 moral and 9 non-moral dilemmas validated and used in a previous rTMS experiment (Tassy et al., 2012). Most of the dilemmas were directly inspired from the battery developed by Greene et al. (2001), translated and adapted to take into account cultural specificities. All were “Sacrificial” moral dilemmas, offering the opportunity to save many people from death (or serious physical consequence) at the cost of one person's life (or serious physical consequences) (Glenn et al., 2010) (e.g., Deadly fumes are rising up in the portion of a hospital where 53 patients are located. Thanks to the ventilation system you can divert the fumes to a room where one patient is sleeping. Is it acceptable to divert the fumes to the room where one patient is sleeping to prevent asphyxiation of 53 patients? Do you divert the fumes to the room where one patient is sleeping to prevent asphyxiation of 53 patients?). Each question was worded so that a positive response favored the survival of the highest number of people (utilitarian response). The non-moral dilemmas required decision making in simple contextual situations with no moral connotation whatsoever. “Appropriate” responses implied the maximization of beneficial overall consequences (e.g., You have to be at a very important meeting at 14 h. You can get there by car or subway. With the subway you will arrive just in time for your meeting. With the car you travel in a more enjoyable way but you will arrive late. Is it acceptable to use the subway instead of your car to be on time at the meeting? Do you use the subway instead of your car to be on time at the meeting?). Because psychopathy is associated to selfishness (Hare, 1999; Mokros et al., 2008), we tested if increasing the personal consequences of the decision would interact with psychopathy traits (Thomas et al., 2011). To do so, in five moral dilemma, the potential victim was supposed to be a family member of the subject (e.g., A train with no brakes is running toward 12 workers. You can divert the train by operating a switch, but it will then go on another track where your ***cousin*** is working.).

Two questions followed the text of each dilemma: one targeting judgment (“Is it acceptable to … in order to …?”) and one targeting choice of action^1^ (“Would you … in order to …?”). To control for any order effect, two types of questionnaires were created: one where the judgment question preceded choice (“Order A” questionnaire), and one where the choice question preceded judgment (“Order B” questionnaire).

### Population

One hundred and two French university students participated to the study (91 females, 22.6 ± 2.3 years old). After receiving oral information about the nature of the experiment, participants completed two anonymous paper questionnaires, one with the moral and non-moral dilemmas and one with the Levenson items. Questionnaires were freely available at the end of a course. Students were free to bring them back later, anonymously, at a dedicated place. They were informed that by accepting to bring anonymously the questionnaires back, they gave their informed consent to participate. Half of the subjects completed a questionnaire “Order A” in which the judgment question preceded the choice question. The other half of each population completed a questionnaire “Order B” in which the choice question preceded the judgment question. The two groups had similar age, gender and LSRP psychopathy score (cf. Table 1).

**Table 1:** **Participant characteristics based on the type of questionnaire (judgment/choice vs. choice/judgment)**.

	**Order A questionnaire judgment/choice (*n* = 51)**		**Order B questionnaire choice/judgment (*n* = 51)**			
	***Mn***	***SD***	***Mn***	***SD***		
Female gender, *n* (%)	47 (92%)		44 (86%)		*p* = 0.525	*X*^2^ = 0.92
Age	22.96	2.27	22.22	2.19	*p* = 0.095	*t* = −1.69
LSRP1	28.61	5.45	29.51	5.42	*p* = 0.404	*t* = 0.84
LSRP2	20.55	3.49	19.71	3.33	*p* = 0.215	*t* = −1.25
LSRP	49.16	7.95	49.22	8.1	*p* = 0.971	*t* = 0.04

*Both groups are identical for studied variables (NS, no significant statistical difference; Mn, mean; SD, standard deviation; LSRP, Levenson Self-Report Psychopathy scale)*.

The whole population showed an average total LSRP score of 49.19 (±7.98), an average LSRP1 score of 29.06 (±5.43), and an average LSRP2 score of 20.13 (±3.42). The two factors of psychopathy were significantly correlated (*r* = 0.613, *p* < 0.001).

It is important to emphasize that in the present study we examine psychopathy as a personality trait that varies within the normal population.

We studied the influence of the level of psychopathic traits on the probability of utilitarian responses to the judgment and choice questions.

### Statistics

For non-moral dilemmas, “appropriate” and “inappropriate” responses were coded 1 and 0, respectively. For moral dilemmas, response to each question was coded 1 if it favored maximizing the good of more people at the expense of very few identified individuals (“utilitarian” response; e.g., sacrificing one person's life to save five), and 0 for the reverse situation.

All statistical analyses were performed using SPSS 17.0 (SPSS Inc., Chicago, IL). For univariate comparisons, we used Student's *t*-test for the means and a Chi-squared test for the percentages.

Multiple logistic regression analyses fitted with the generalized estimating equations method to account for the within-subject correlation (Koenigs et al., 2007) were conducted using each response to a dilemma as the dependent variable and entering total psychopathy score, sex, affective proximity of the victim, and the type of questionnaire as predictors. Sex was entered as control variable in all analyses because it has been reported to significantly influence moral decision making (Fumagalli et al., 2010). An additional regression was conducted in which both factors of psychopathy (LSRP1 and LSRP2) were simultaneously entered as predictors in place of the total psychopathy score. When the level of psychopathic traits had a significant effect, first order interaction between this level and other potential predictors were systematically studied.

## Results

### Influence of studied variables, on non-moral dilemma evaluation

No variable significantly predicted responses to non-moral dilemmas either for the judgment or the choice question. Psychopathy traits thus do not influence response in easy non-moral decision making situations.

### Influence of studied variables on moral dilemma evaluation

In the case of moral dilemmas, a high level of psychopathy traits, male sex, and affective distance with the victim significantly predicted utilitarian response to the choice question. For the judgment question, only the male sex significantly predicted utilitarian response, but neither the level of psychopathy traits nor the affective distance with the victim.

Order of the question (judgment before choice or vice-versa) did not influence the response for the judgment and for the choice (cf. Table 2).

**Table 2:** **Regression analyses demonstrating associations between utilitarian responses to moral dilemma and predictors**.

	**Judgement question**		**Choice question**	
**Predictors**	**β-values**	***p***	**β-values**	***p***
Male sex	0.58	0.017	0.57	0.019
Affective proximity	−0.08	0.470	−1.11	<0.001
Order of question	0.03	0.869	−0.01	0.964
**Total psychopathy**	**0.01**	**0.945**	**0.19**	**0.048**
^*^LSRP1	**0.09**	**0.601**	**0.40**	**0.029**
^*^LSRP2	−**0.15**	**0.603**	−**0.18**	**0.534**

*Note: The beta values are from multiple regression models predicting utilitarian response to moral dilemma from total psychopathy score (per 10-point increase), sex, affective proximity of the victim, and the type of questionnaire. Numbers indicate standardized beta (β)*.

* *Beta values for the LSRP1 and LSRP2 are from multiple regression models using sex, affective proximity, type of questionnaire, and both psychopathy factors (per 10-point increase) as predictors. Bold style is for the variables of interest*.

### Influence of both factors of psychopathy on dilemma evaluation

Only higher LSRP1 (affective and interpersonal dimension) score significantly predicted a bias toward utilitarian response to the choice question (cf. Table 2).

### Influence of interaction between psychopathy traits and other significant predictors

We did not find any interaction with total psychopathy traits (^*^sex β = 0.18; *p* = 0.532; ^*^affective proximity β = −0.14; *p* = 0.370) or LRSP1 (^*^sex β = 0.22; *p* = 0.742; ^*^affective proximity β = −0.16; *p* = 0.388) that significantly predicted utilitarian responses to the choice question. Increasing affective proximity of the victim (i.e., stronger personal consequences) did not interact with psychopathy traits' influence on moral choice responses.

## Discussion

A high level of psychopathy traits does not predict utilitarian judgment during moral dilemmas evaluation, but it predicts utilitarian response in the case of choice. This effect is observed more specifically for the interpersonal-affective characteristics of psychopathy as measured by the LSRP1. This suggests that while the evaluative moral judgment of individuals with a high level of psychopathic traits (HP) remains identical to the judgment of individuals with normal/low level of psychopathic traits, they are more likely to make an effective choice decision that would inflict suffering or death to an individual for the greater welfare of more people. A high level of psychopathic traits thus influences the choice of hypothetical action endorsement embedded in a moral dilemma, but not moral judgment. Consistent with what is known in the case of patients with VMPFc lesions who exhibit emotional deficits and endorse utilitarian responses to moral dilemmas (Koenigs et al., 2007), we found that, in non-clinical individuals, scoring higher on a general measure of psychopathic traits and a measure of psychopathic traits targeting shallow affects and VMPFc hypofunctioning (Lotze et al., 2007) predicts utilitarian action endorsement preferences.

This may help explain the discrepant results of previous studies on moral dilemma evaluation in psychopathic individuals. Some studies indeed claimed that the ability to evaluate moral dilemmas is preserved in psychopathy (Cima et al., 2010), while others claimed that this ability is altered (Koenigs et al., 2012). Several factors are potentially responsible for this variability. It has been proposed that differences in the population from which the subjects were drawn may explain the discrepancy between these studies as most studies have sampled directly from a psychiatric population or a population of criminal offenders. Individuals diagnosed with psychopathy may be highly motivated to report in a manner that they believe will make them seem like an “average” individual because, among other reasons, they may be concerned that their responses may have consequences for their treatment or incarceration (Bartels and Pizarro, 2011). Another potential factor is the scale used to measure psychopathy (Koenigs et al., 2011). However, in the present study we used a scale that has been used in previous studies on moral decision making (Glenn et al., 2010) and shows a good concordance with the PCL-R (Brinkley et al., 2001) and SRPIII scales (Williams et al., 2003) used in other studies on moral evaluation in psychopathy (Cima et al., 2010; Bartels and Pizarro, 2011).

On the basis of our results, we propose that some discrepancies in the results of previous studies could originate from the use of moral dilemmas that differ on other dimensions, including the wording or structure of the evaluation question (O'Neill and Petrinovich, 1998; O'Hara et al., 2010), which may modify responses because they do not target the same evaluative psychological processes. Indeed, if one goes into details of the experimental procedures, responses were unaltered only when the question was worded as an evaluative judgment (“*Is it moral for you to …* ”) (Cima et al., 2010). By contrast, psychopaths showed an altered response when the question was worded as a behavioral choice (“*Would you … in order to …* ?”) (Bartels and Pizarro, 2011; Koenigs et al., 2012). At the cerebral level right dorso-lateral prefrontal cortex (DLPFc) disruption alters moral judgment but not choice, which suggest that this structure is required to process allocentric integration of contextual information during moral judgment (Frith and de Vignemont, 2005; Tassy et al., 2012). On the contrary, a high level of psychopathy traits characterized by VMPFc dysfunction (Blair, 2007; Lotze et al., 2007; Koenigs, 2012), alters moral choice but not moral judgment. This suggests that moral choice of action mostly involves VMPFc. In the same line, Glenn et al. (2009) found that higher psychopathy scores are associated with reduced activity in VMPFc during moral choice of action (“*Would you … in order to*”). By contrast, as previously hypothesized (Tassy et al., 2009). The same study reported that higher psychopathy scores are also associated with increased activity in the rDLPFc during moral dilemma resolution. Individuals with a high level of psychopathic traits, because they lack some emotional reactions (Blair, 2007), may thus rely on allocentric judgment of the situation to make a choice decision.

As expected from results of previous psychological studies, affective proximity of the potential victims influences responses toward less utilitarianism (O'Neill and Petrinovich, 1998; Thomas et al., 2011). This is true for both judgment and choice, but seems to be stronger for the choice [β choice = 1.11, 95% confidence interval (0.87; 1.35) > β judgment = 0.01 (−0.14; 0.30)], which is coherent with recent studies (Kurzban et al., 2012; Tassy et al., 2013). A potential explanation could be that implication of a kin has strong personal consequences (Thomas et al., 2011), and personal consequences influence more action choice than abstract judgment (Sood and Forehand, 2005). We didn't find any interaction between level of psychopathy traits' influence and affective proximity. The response bias toward more utilitarianism of individuals with higher level of psychopathic traits is not influence by the affective proximity of the victim. It thus suggests that the response bias toward more utilitarianism of individuals with higher level of psychopathic traits is not influence by stronger personal consequences. This may appear opposite to selfishness theoretically associated with psychopathy (Campbell et al., 2009). A reason should be that psychopathy results from a strong default of emotional reaction to other distress. The distress of others emotionally arouses individuals with a low level of psychopathy traits. When the other is affectively proximate it potentiates this reaction. It results in decreased utilitarianism in responses (to reduce other's distress). Individuals with high level of psychopathic traits lack emotional reaction to the distress of others (Lotze et al., 2007), thus this reaction cannot be potentiated.

As emphasized by Sood and Forehand (Sood and Forehand, 2005), compared to judgment, choice elicits a greater degree of self-referent processing. Choice differs from judgment in agency because it implies projecting oneself into a situation of direct interaction using an egocentric frame of reference with potential self-relevant consequences. It thus generates strong emotional reactions and is also largely influenced by self-interest rational maximization. By contrast, judgment relies on an evaluation of the situation from a more allocentric perspective as defined by Frith and de Vignemont (Frith and de Vignemont, 2005). It is thus less influenced by self-interest maximization. Responses during moral dilemma evaluation indeed differ depending on whether evaluators are agents in the scenario rather than observers (Nadelhoffer and Feltz, 2008) and participants' intuition about their own or other's moral transgression activate distinct brain regions (Berthoz et al., 2006). It may explain the variation of the degree of utilitarianism in various dilemma responses where the dilemma induces an abstract judgment (reaction to moral violation by another person) or a choice of action (i.e., from a first person perspective) (Monin et al., 2007). This could also explain why some studies reported that people acknowledge moral norms and make appropriate moral judgment but fail to act in accordance with them, illustrating a capacity for “moral hypocrisy” (Batson et al., 1997).

Such a discrepancy was already noted in the field of developmental psychology (Blasi, 1980). In line with such a view, the results from the present study give further original experimental support to the notion that choice and judgment during moral dilemma evaluation are partially distinct psychological processes (Tassy et al., 2012). In itself, this may be sufficient to raise a methodological concern in the study of moral cognition, and propose that choice of action and evaluative judgment should be conceptualized and tested separately. It would help understanding exactly which moral processes are affected in psychopathy, and could be used as a tool to test potential therapeutic effect (Krueger et al., 2012). More generally, it would improve in-depth understanding of moral motivation and cognition.

Overall, our empirical data nourish the debate on the role of our emotions and feelings about particular actions and outcomes as a source of our moral judgment and moral behavior (Moll et al., 2005, 2007; Shenhav and Greene, 2010) by revealing different patterns of moral evaluation in HP compared to Control normal population individuals, at least for a particular type of decision. Besides this, it brings a potential methodological answer to the variability observed in previous studies exploring moral evaluation in psychopathy. Overall, it highlights the fact that moral cognition consists of many levels of complexity that need to be better understood and taken into account.

## Funding

No current external funding sources for this study.

### Conflict of interest statement

The authors declare that the research was conducted in the absence of any commercial or financial relationships that could be construed as a potential conflict of interest.

## Footnotes

^1^Our study is not immune to a usual critique that can be raised in this kind of setting involving moral dilemmas: what we consider here as an action is obviously what the participants think their action could be if they were to make the decision in real life. For ethical reasons, using questionnaires is as far as we can go given the life and death nature of the dilemmas.
